# Perceived urban environment attributes and obesity indices in adults: an 8-Nation study from Latin America

**DOI:** 10.1038/s41598-022-24209-2

**Published:** 2022-11-15

**Authors:** Gerson Ferrari, Claudia Alberico, Adilson Marques, Irina Kovalskys, Georgina Gómez, Attilio Rigotti, Lilia Yadira Cortés, Martha Yépez García, Rossina G. Pareja, Marianella Herrera-Cuenca, Clemens Drenowatz, Ana Carolina B. Leme, Carlos Cristi-Montero, Roberto Fernandes da Costa, Claudio Farías‑Valenzuela, Mauro Fisberg

**Affiliations:** 1 Facultad de Ciencias de la Salud, Universidad Autónoma de Chile, Dirección: Av. Pedro de Valdivia 425, Santiago, Providencia Chile; 2 North Carolina Central University (NCCU), Julius L. Chambers Biomedical and Biotechnology Research Institute (JLC-BBRI), Durham, NC USA; 3 CIPER, Faculdade de Motricidade Humana, Universidade de Lisboa, Lisbon, Portugal; 4 Faculdade de Medicina, ISAMB, Universidade de Lisboa, Lisbon, Portugal; 5 Carrera de Nutrición, Facultad de Ciencias Médicas, Pontificia Universidad Católica Argentina, Buenos Aires, Argentina; 6 Departamento de Bioquímica, Escuela de Medicina, Universidad de Costa Rica, San José, Costa Rica; 7 Centro de Nutrición Molecular y Enfermedades Crónicas, Departamento de Nutrición, Diabetes Y Metabolismo, Escuela de Medicina, Pontificia Universidad Católica, Santiago, Chile; 8 Departamento de Nutrición Y Bioquímica, Pontificia Universidad Javeriana, Bogotá, Colombia; 9 Colégio de Ciencias de la Salud, Universidad San Francisco de Quito, Quito, Ecuador; 10 Instituto de Investigación Nutricional, La Molina, Lima Peru; 11 Centro de Estudios del Desarrollo, Universidad Central de Venezuela (CENDES-UCV)/Fundación Bengoa, Caracas, Venezuela; 12 Division of Physical Education, University of Education Upper Austria, 4020 Linz, Austria; 13 Centro de Excelencia em Nutrição e Dificuldades Alimentaes (CENDA), Instituto Pensi, Fundação José Luiz Egydio Setubal, Hospital Infantil Sabará, São Paulo, Brazil; 14 IRyS Group, Physical Education School, Pontificia Universidad Católica de Valparaíso, Valparaíso, Chile; 15 Physical Education Department, Research Group in Physical Activity and Health, Federal University of Rio Grande do Norte, Natal, Brazil; 16 Instituto del Deporte, Universidad de las Americas, 9170022 Santiago, Chile; 17 Departamento de Pediatria da, Universidade Federal de São Paulo, São Paulo, Brazil

**Keywords:** Risk factors, Environmental impact, Cardiovascular diseases, Nutrition disorders

## Abstract

This study examines the associations between perceived urban environment attributes and obesity indices by country using data from an eight-nation study from Latin America. The data were collected from 8185 adults. The Neighbourhood Environment Walkability-abbreviated scale was used to assess perceived urban environment attributes. Obesity indices considered were body mass index, waist circumference, neck circumference, a body shape index and waist-to-height ratio. The perception of a more and better land use mix-diversity (β − 0.44; 95% CI − 0.59, − 0.28), traffic safety (− 0.39; − 0.66, − 0.12), and safety from crime (− 0.36; − 0.57, − 0.15) was associated with lower body mass index across the entire sample. Land use mix-diversity (− 1.21; − 1.60, − 0.82), street connectivity (− 0.26; − 0.37, − 0.15), and traffic safety (− 0.79; − 1.47, − 0.12) were negatively associated with waist circumference. Land use mix-diversity (− 0.11; − 0.20, − 0.03), land use mix-access (− 0.23; − 0.34, 0.12), walking/cycling facilities (− 0.22; − 0.37, − 0.08), and safety from crime (− 0.27; − 0.42, − 0.12) were negatively associated with neck circumference. No associations between perceived urban environment attributes and a body shape index were found. Land use mix-diversity (− 0.01; − 0.02, − 0.01), aesthetics (− 0.02; − 0.03, − 0.01), and safety from crime (− 0.02; − 0.04, − 0.01) were associated with waist-to-height ratio. Environmental interventions involving urban environment attributes are associated with obesity indices and, therefore, may help decrease the prevalence of overweight and obesity.

## Introduction

Overweight and obesity are important health problems worldwide and puts a considerable burden on public health. Numerous studies have reported that being overweight or obese has a strong negative health impact^1–3^. Approximately 39% of adults globally are overweight or obese^4^. Furthermore, alarming rates of overweight and obesity (> 60%) have been reported in Latin America^5^.

In the past 30 years, Latin America experienced a fast shift in populations from rural to urban settings; currently, around 80% of the Latin America population live in cities, contributing to the formation of mega cities^6^. Also, during the same period, Latin America faced noteworthy demographic, epidemiological and socioeconomic development and lifestyle changes resulting in distinct urban characteristics than North American and Western European countries^6^. Furthermore, the increased and unregulated expansion of urban environments has resulted in an ecosystem that courage’s unhealthy eating and an inactive lifestyle^7–9^.

Ecological models indicate that multiple levels of behavior, including inter-personal/social factors, institutional, community, built environment, and policies that could be tackled to prevent inappropriate lifestyle^10,11^. Despite public health efforts to reduce overweight and obesity through diet and physical activity, rates remain high, prompting a search for population-wide strategies to help curb these conditions. One approach that is gaining interest among public health professionals and urban planners is to redesign the built environment to offer more opportunities for physical activity and healthy eating^12^. This concept suggests that the built environment and lifestyle of urban residents affects their health status and one of the assumptions is that unexpected health problems have appeared due to rapid global urbanization. As a result of increasing urbanization, people have no choice but to live in confined spaces, and in addition, environments that increase the weight of urban residents have appeared, and car usage has increased as the distance between residences and workplaces has widened^13,14^.

In Latin America, urban environment attributes were mostly considered in relation to physical activity^15,16^. On the other hand, there is limited evidence on the association between perceived urban environment attributes and obesity indices^9,17,18^. Epidemiologic studies^17^ and systematic reviews^19–21^ indicate that the findings of these associations are varied. Although some of the apparent discrepancies could be due to diversity of procedures engaged, the main issue that may clarify this variety in results is the limited variability. Almost all studies of association between the perceived urban environment attributes and obesity indices have been conducted within individual countries and most commonly in a single city^22^. An example of a large, scale multi-country study is the International Physical Activity Network (IPEN) study of adults from 17 cities in 12 countries, including three cities from Latin America (Bogota [Colombia], Curitiba [Brazil], Cuernavaca [Mexico]). The results indicated significant associations of traffic, proximity to several local destinations, and safety from crime with body mass index^17^.

Furthermore, environmental influences on health behavior may be dependent on the type of measurement of the physical environment; objective measures are often regarded as superior to subjective reports, however, the form in which a person perceives their environment has a significant impact on their motivations to be active^23,24^. To our knowledge, none of these existing Latin American studies, however, have identified associations between perceived urban environment attributes and anthropometric indices of risk factors to non-communicable chronic diseases from multiple countries using comparable methods in a representative sample of the urban population.

In addition to the body mass index, several studies have demonstrated the association of anthropometric indices such as neck and waist circumferences, waist-to-height ratio and body shape index, with risk factors for chronic diseases and mortality^25–28^. Although BMI is the best-known anthropometric index and is used as a way of estimating overweight and obesity, it does not provide any information on the distribution of body fat^29^. In addition, other indices have shown better results than BMI for estimating risk for chronic non-communicable diseases associated with central adiposity^30–32^. Thus, the present study's purpose was to examine the associations between perceived urban environment attributes and obesity indices in adults by country and overall data from eight-nation study from Latin America.

## Methods

### Study design and participants

The Latin American Study of Nutrition and Health (Estudio Latinoamericano de Nutrición y Salud—ELANS) is a large cross-sectional study that uses comparable methods across 8 Latin America countries (Argentina, Brazil, Chile, Colombia, Costa Rica, Ecuador, Peru, and Venezuela) and focuses on the urban population. Data collection occurred between 2014 and 2015. The ELANS protocol was approved by the Western Institutional Review Board (no 20140605) and registered in Clinicaltrials.gov (no NCT02226627). Also, the local ethics institutional review boards from each country approved the study. Participants provided written informed consent/assent before data collection and this study was performed in accordance with relevant guidelines and regulations. ELANS protocol details have been published elsewhere^16,33^.

Participants were selected using a random complex, multistage sampling frame with a random selection of Primary Sampling Unit (PSU) areas (e.g., counties, municipalities, neighborhoods, residential areas). A sample size proportional to population weight was used for the selection of PSU. In this circumstance, a simple random sampling of N with replacement was executed to adhere to the principle of statistical independence of the selection of the areas included in the PSU sample. Within each of the areas included in the PSU distribution, a representative set of Secondary Sampling Units was randomly identified using the probability proportional to size method.

Households were selected based on a four-step, systematic randomization procedure by establishing a selection interval (k): (a) the total urban population was used to proportionally describe the main regions and to select cities representing each region; (b) the sampling points (survey tracts) of each city were randomly designated, and (c) clusters of households were selected from each sampling unit; (d) the designated respondent within each household was selected using the birthday method.

The sampling size was calculated with a confidence level of 95% a maximum error of 3.5%, and a survey design effect of 1.75, resulting in a required sample size of 9090. The minimum sample sizes required per strata (sex, age group, and socioeconomic level) was performed for each country. The overall sample consisted of 9218 (4809 women) participants aged 15–65 years from the urban population of eight Latin America countries. Details on study design, recruitment methods and response rates by country, region and city have been published previously^16,34,35^. For this analysis, those aged 15–17 years were excluded as the perception of adolescents is different from that of adults, due to responsibilities and legal abilities in these Latin American countries. The final sample, therefore consisted of 8185 participants between 18 and 65 years who had complete data on perceived urban environment attributes and obesity indices.

### Perceived urban environment attributes

The abbreviated Neighbourhood Environment Walkability Scale (NEWS-A) is a reliable and validated self-report instrument that captures perceived urban environment attributes^36^. The validated NEWS-A has been previously translated into Spanish and was adapted for use in Latin America to assess perceived urban environment attributes^36–38^.

Cerin et al.^36^ reported the following subscales that were used for the ELANS multi-country pooled analyses: land use mix–diversity, use mix-access, street connectivity, walking/cycling facilities, aesthetics, traffic safety, and safety from crime. Scales were scored in a direction consistent with higher scores reflecting higher perceived urban environment attributes and more safety, with individual items reversed when necessary. The overall average score of land use mix-diversity (1 to 5-min walking distance [coded as 5, indicative of high walkability] to > 30-min walking distance [coded as 1, indicative of low walkability]) was 2.8. The overall scores were 3.0 for land use mix-access, 2.8 for street connectivity, 2.8 for walking/cycling facilities, 2.6 for aesthetics, 2.6 for safety from traffic, and 2.5 for safety from crime (1 = strongly disagree to 4 = strongly agree; higher scores reflect more activity friendliness). Details about all items captured, internal consistency between each scale and scoring details are described elsewhere^16,39^. The average score of each scale and Cronbach’s alpha values for NEWS-A subscales have also been published elsewhere^16,34^.

### Obesity indices

All participating countries performed anthropometric measurements of body weight, height, waist circumference, and neck circumference according to standardized procedures^40^. Body weight was measured to the nearest 0.1 kg using a portable scale (Seca Corporation, Hamburg, Germany) after all outer clothing, heavy pocket items, shoes, and socks were removed. Height was measured to the nearest 0.5 cm without shoes using a Seca 213 portable stadiometer (Seca Corporation, Hamburg, Germany) with the participant’s head in the Frankfurt Plane^41^. Body mass index (kg/m^2^) was calculated (weight [kg]/height [m^2^]). The body mass index standard deviation (SD) scores of participants between 18–19 years old were derived using the age- and sex-specific World Health Organization (WHO) growth reference for school-aged youth which were classified into four categories: underweight (< − 2SD), eutrophic (− 2SD ≥ to ≤ 1SD), overweight (1SD > to ≤ 2SD), and obese (> 2SD)^42^. Adults (≥ 20 years old) were also categorized as underweight (< 18.5), eutrophic (18.5 to ≤ 24.9), overweight (≥ 25.0 to ≤ 29.9), or obese (≥ 30.0)^43^.

Waist circumference (cm) was measured midpoint between the lower coastal ridge and the upper margin of the superior iliac crest, using a flexible plastic tape^44^. Categorization was stratified by sex, in accordance with the standardized protocol; central obesity for female participants was defined as > 88 cm and > 102 cm for male participants^45^.

Neck circumference (cm) was measured at the point just below the larynx (thyroid cartilage) and perpendicular to the long axis of the neck (with the tape line in the front of the neck at the same height as the tape line at the back of the neck) using an inelastic tape measure^46^. Neck circumference (cm) was categorized as abnormal if circumference was > 39 cm for men and > 35 cm women^47^. These cut-points are probably the best to determine individuals with central obesity and metabolic syndrome^47^.

A body shape index was calculated using the following equations: waist circumference (m)/body mass index (kg/m^2^)^2∕3^ ∗ height(m)^1/2^. A body shape index has been shown to be associated with cardiovascular risk factors, mortality, and cardiovascular events in several ethnic groups^27,48–50^. A body shape index expresses the excess risk from high waist circumference in a convenient form that is complementary to body mass index and to other known risk factors^48^. A body shape index was categorized as below threshold if result was < 0.0812^51^.

Waist-to-height ratio was computed as waist circumference/height both in centimeters^30^. In order to assign severity grades to the screening parameter waist-to-height ratio, a normal ratio was considered if waist-to-height ratio ≤ 0.5, risk if waist-to-height ratio > 0.5 to ≤ 0.6 and abnormal if waist-to-height ratio > 0.6^30^.

### Correlates

Age, sex, marital status, work status, socioeconomic level, and energy intake were assessed using standard questionnaires that were completed during face-to-face interviews and included as covariates in all statistical models. Marital status was classified as married and not-married (single, widowed or divorced). Work status was categorized as working (part time and full time), and not-working (student, unemployed, retired, and other). Socioeconomic level data was divided into three strata (low, medium, and high) based on the national indexes used in each country. Dietary intake data was obtained from two in-person 24 h dietary recall interviews using the automated multiple-pass method^52,53^. Foods and beverages were converted into energy and nutrients using the Nutrition Data System for Research Software (NDS-R version 2013)^54^. Energy intake was also used as potential confounder to evaluate the associations between perceived urban environment attributes and obesity indices. Detailed information can be found in a previous publication^16,55^.

### Statistical analysis

Participants who provided complete data for the variables of interest were included in this study. Weighting was done according to sociodemographic characteristics, sex, socioeconomic level, and country^16^.

Kolmogorov–Smirnov test was used to check the normal distribution of the data. Descriptive data were reported as means, standard deviation, frequency, and percentage according to country. Internal consistency of the environment attributes characteristics’ scales was examined using Cronbach’s alpha.

Linear regression models (b-coefficient, confidence interval 95%: 95% CI) were estimated using unstandardized coefficient values to estimate the associations of perceived urban environment attributes characteristics with each obesity indices (body mass index, waist circumference, neck circumference, a body shape index, and waist-to-height ratio). The models were adjusted for age, sex, marital status, work status, socioeconomic level, and energy intake. Separate regression models were then run for each country. In this study we used the “enter” method for the regression analysis with all variables in a block entered in a single step as predictors. Results were computed for the overall sample and by country. All statistical analyses were conducted using SPSS V28 software (SPSS Inc., IBM Corp., Armonk, New York, NY, USA). A significance level of p < 0.05 was adopted.

### Ethics approval and consent to participate

Ethical approval was provided by the Western Institutional Review Board (#20140605), and by the ethical review boards of the participating institutions. ELANS is registered at Clinical Trials #NCT02226627. Written informed consent/assent was obtained from all individuals, before commencement of the study. This study was performed in accordance with relevant guidelines and regulations.

## Results

Participant characteristics per county and overall are presented in Table 1. Among 8185 adult participants, 4341 (53.0%) were women, with a mean age of 37.4 (SD: 13.3). Overall, 51.2% of participants were married, 57.0% working, and 52.0% had low socioeconomic level. The mean values of energy intake, body mass index, waist circumference, neck circumference, a body shape index, and waist-to-height ratio were 1982.4 (SD: 621.5) kcal/day, 27.3 (SD: 5.6) kg/m^2^, 89.3 (SD: 14.1) cm, 35.8 (SD: 4.1) cm, 0.077 (SD: 0.006), and 0.62 (SD: 0.08), respectively. Furthermore, 62.4% of participants were overweight or obese, 33.8%, 35.0% and 32.7% were above the waist circumference, neck circumference and a body shape index threshold. Finally, 41.8% and 54.1% of participants were classified as risk and abnormal of waist-to-height ratio.

**Table 1:** Demographic characteristics (mean [SD] or %) and obesity indices of participants in overall and by country.

Variables	Overall	Argentina	Brazil	Chile	Colombia	Costa Rica	Ecuador	Peru	Venezuela
**Sample size (n)**	8185	1137	1803	770	1105	710	668	979	1013
**Age [years, mean (SD)]**	37.4 (13.3)	38.4 (13.2)	37.9 (13.1)	38.0 (13.4)	38.2 (13.9)	37.0 (13.2)	35.9 (13.4)	35.9 (12.9)	36.5 (13.2)
**Sex (%)**									
Men	47.0	43.6	46.3	47.3	48.2	48.9	49.0	46.3	48.4
Women	53.0	56.4	53.7	52.7	51.8	51.1	51.0	53.7	51.6
**Marital status (%)**									
Married	51.2	52.9	49.7	50.0	48.3	50.4	55.7	57.3	47.3
Not-married	48.8	47.1	50.3	50.0	51.7	49.6	44.3	42.7	52.7
**Work status (%)**									
Working	57.0	66.2	62.0	57.5	49.0	47.6	49.7	55.1	59.1
Not-working	43.0	33.8	38.0	42.5	51.0	52.4	50.3	44.9	40.9
**Socioeconomic level (%)**									
Low	52.0	47.4	46.2	46.0	63.0	33.0	51.2	47.6	77.9
Medium	38.4	47.2	45.2	44.6	31.6	53.2	35.9	31.6	17.2
High	9.6	5.4	8.6	9.4	5.4	13.8	12.9	20.7	4.9
Energy intake [kcal/day, mean (SD)]	1982.4 (621.5)	2163.3 (676.6)	1820.5 (599.8)	1724.1 (551.9)	2115.8 (583.9)	1882.8 (627.3)	2215.1 (611.9)	2108.7 (561.0)	1904.1 (565.6)
**Body mass index**									
Kg/m^2^ [mean (SD)]	27.3 (5.6)	27.5 (5.9)	27.0 (5.6)	28.5 (5.4)	26.1 (5.0)	28.1 (6.2)	27.2 (5.4)	27.1 (4.9)	27.7 (5.8)
Categorical (%)									
Underweight	2.6	2.4	3.5	0.3	3.6	2.1	3.0	1.7	2.9
Eutrophic	35.0	36.3	36.3	28.1	42.6	32.2	33.1	34.7	32.1
Overweight	35.6	33.0	34.2	38.2	35.7	33.6	37.6	39.8	35.1
Obese	26.8	28.3	26.0	33.4	18.1	32.1	26.3	23.8	29.9
**Waist circumference**									
Cm [mean (SD)]	89.3 (14.1)	89.6 (15.5)	88.4 (14.3)	93.4 (14.1)	85.9 (12.8)	93.0 (15.2)	88.7 (11.9)	88.4 (12.1)	90.0 (14.2)
Categorical (%)									
Below threshold	66.2	64.6	68.2	57.3	76.0	58.3	69.2	67.2	63.5
Above threshold	33.8	35.4	31.8	42.7	24.0	41.7	30.8	32.8	36.5
**Neck circumference**									
Cm [mean (SD)]	35.8 (4.1)	35.7 (4.0)	34.9 (4.5)	37.5 (3.9)	35.4 (3.5)	36.9 (3.9)	35.3 (3.7)	35.6 (3.6)	36.4 (4.2)
Categorical (%)									
Below threshold	65.0	65.0	71.7	46.8	73.5	53.9	71.0	68.3	58.6
Above threshold	35.0	35.0	28.3	53.2	26.5	46.1	29.0	31.7	41.4
**A body shape index**									
Z score [mean (SD)]	0.077 (0.006)	0.077 (0.006)	0.077 (0.006)	0.077 (0.006)	0.077 (0.006)	0.077 (0.006)	0.077 (0.006)	0.077 (0.005)	0.077 (0.006)
Categorical (%)									
Below threshold	67.3	66.4	68.2	63.7	67.5	65.5	66.6	70.3	68.0
Above threshold	32.7	33.6	31.8	36.3	32.5	34.5	33.4	29.7	32.0
**Waist-to-height ratio**									
Cm (SD)	0.62 (0.08)	0.63 (0.08)	0.60 (0.08)	0.64 (0.07)	0.60 (0.07)	0.63 (0.09)	0.62 (0.07)	0.62 (0.06)	0.62 (0.08)
Categorical (%)									
Normal	4.1	1.7	7.7	1.0	6.8	2.5	1.8	1.1	5.1
Risk	41.8	41.4	45.3	34.8	46.6	39.9	40.0	41.7	39.1
Abnormal	54.1	56.9	47.0	64.2	46.6	57.6	58.2	57.2	55.8

*SD* standard deviation.

Overall, the perception of more and better land use mix-diversity (β: – 0.44; 95% CI − 0.59; − 0.28), traffic safety (β: − 0.39; 95% CI − 0.66; − 0.12), and safety from crime (β: − 0.36; 95% CI − 0.57; − 0.15) was negatively associated with body mass index (kg/m^2^). Some distinct associations between perceived urban environment attributes and body mass index by country were detected. Argentina was the country with the strongest associations between perceived urban environment attributes (land use mix-diversity: β: − 0.45; 95% CI − 0.88; − 0.01; aesthetics: β: − 0.60; 95% CI − 1.13; − 0.08; safety from traffic: β: − 1.04; 95% CI − 1.73; − 0.35) and body mass index. On the other hand, there were no associations between any perceived urban environment attributes and body mass index in Chile, Colombia, Costa Rica, Ecuador, and Peru (Table 2).

**Table 2:** Linear regression models (unstandardized β, 95%CI) for perceived urban environment attributes and body mass index (kg/m^2^).

Independent variables	Overall	Argentina	Brazil	Chile	Colombia	Costa Rica	Ecuador	Peru	Venezuela
	β (95% CI)	β (95% CI)	β (95% CI)	β (95% CI)	β (95% CI)	β (95% CI)	β (95% CI)	β (95% CI)	β (95% CI)
Land use mix-diversity (score 1–5)^1^	− 0.44* (− 0.59; − 0.28)	− 0.45* (− 0.88; − 0.01)	− 0.36* (− 0.69; − 0.04)	− 0.55 (− 1.20; 0.09)	− 0.05 (− 0.46; 0.35)	− 0.51 (− 1.08; 0.06)	− 0.55 (− 1.19; 0.07)	− 0.31 (− 0.78; 0.15)	− 0.24 (− 0.67; 0.19)
Land use mix-access (score 1–4)^1^	0.13 (− 0.15; 0.42)	0.01 (− 0.80; 0.80)	− 0.18 (− 0.84; 0.47)	− 0.22 (− 1.11; 0.65)	0.16 (− 0.64; 0.97)	0.38 (− 0.64; 1.42)	0.60 (− 0.51; 1.72)	− 0.19 (− 0.94; 0.56)	− 1.20* (− 2.02; − 0.38)
Street connectivity (score 1–4)^2^	0.15 (− 0.05; 0.37)	0.20 (− 0.35; 0.76)	0.41 (− 0.02; 0.86)	0.19 (− 0.38; 0.77)	− 0.31 (− 0.95; 0.32)	− 0.01 (− 0.80; 0.78)	− 0.11 (− 1.00; 0.77)	− 0.36 (− 0.98; 0.26)	− 0.05 (− 0.69; 0.59)
Walking/cycling facilities (score 1–4)^1^	− 0.02 (− 0.21; 0.17)	0.25 (− 0.41; 0.91)	0.38 (− 0.07; 0.84)	0.20 (− 0.43; 0.84)	0.24 (− 0.33; 0.81)	0.20 (− 0.39; 0.81)	0.34 (− 1.25; 0.56)	− 0.05 (− 0.50; 0.40)	− 0.46* (− 0.68; − 0.24)
Aesthetics (score 1–4)^1^	− 0.05 (− 0.22; 0.11)	− 0.60* (− 1.13; − 0.08)	− 0.15 (− 0.50; 0.18)	− 0.31 (− 0.82; 0.18)	0.06 (− 0.46; 0.58)	− 0.10 (− 0.75; 0.54)	− 0.50 (− 0.75; 0.65)	0.13 (− 0.33; 0.60)	0.15 (− 0.33; 0.64)
Safety from traffic (score 1–4)^2^	− 0.39* (− 0.66; − 0.12)	− 1.04* (− 1.73; − 0.35)	− 0.34 (− 0.96; 0.27)	0.10 (− 0.61; 0.82)	− 0.36 (− 1.05; 0.32)	− 0.27 (− 1.19; 0.64)	− 0.33 (− 1.28; 0.62)	− 0.34 (− 1.10; 0.41)	− 0.09 (− 0.96; 0.77)
Safety from crime (score 1–4)^1^	− 0.36* (− 0.57; − 0.15)	− 0.51 (− 1.14; 0.12)	− 0.80* (− 1.27; − 0.34)	− 0.54 (− 1.18; 0.09)	− 0.35 (− 0.93; 0.229	− 0.16 (− 0.93; 0.59)	− 0.86* (− 1.67; − 0.05)	− 0.48 (− 1.14; 0.18)	0.45 (− 0.16; 1.07)

Linear regression models adjusted for age, sex, marital status, work status, socioeconomic level, and energy intake.

*Indicates statistically significant associations (p < 0.05).

*CI* confidence interval;

^1^Higher scores indicate perception of higher land use mix-diversity, higher land use mix-access, more walking/cycling facilities, better aesthetics, and more safety from crime.

^2^4-point scale: strongly disagree (1), disagree (2), agree (3), strongly agree (4).

Overall, land use mix-diversity (β: − 1.21, 95% CI − 1.60; − 0.82), street connectivity (β: − 0.26, 95% CI − 0.37; − 0.15) and traffic safety (β: − 0.79, 95% CI − 1.47; − 0.12) were negatively associated with waist circumference (cm). Distinct associations by country were detected between perceived urban environment attributes and waist circumference (cm). Brazil was the country with the strongest associations between perceived urban environment attributes (land use mix-diversity: β: − 1.11; 95% CI − 1.93; − 2.92; land use mix-access: β: − 2.29; 95% CI − 3.95; − 0.63; street connectivity: β: − 0.56; 95% CI − 0.68; − 0.44; safety from traffic: β: − 1.40; 95% CI − 1.67; − 1.13) and waist circumference (cm). There were no associations between any perceived urban environment attributes and waist circumference in Chile, Colombia, Costa Rica, Ecuador, and Peru (Table 3).

**Table 3:** Linear regression models (unstandardized β, 95% CI) for perceived urban environment attributes and waist circumference (cm).

Independent variables	Overall	Argentina	Brazil	Chile	Colombia	Costa Rica	Ecuador	Peru	Venezuela
	β (95% CI)	β (95% CI)	β (95% CI)	β (95% CI)	β (95% CI)	β (95% CI)	β (95% CI)	β (95% CI)	β (95% CI)
Land use mix-diversity (score 1–5)^1^	− 1.21* (− 1.60; − 0.82)	− 1.93* (− 3.06; − 0.81)	− 1.11* (− 1.93; − 2.92)	− 0.76 (− 2,46; 0.92)	− 0.53 (− 1.57; 0.51)	− 0.92 (− 2.34; 0.49)	− 0.44 (− 1.86; 0.96)	− 0.65 (− 1.81; 0.50)	− 0.577 (− 1.65; 0.50)
Land use mix-access (score 1–4)^1^	0.39 (− 0.33; 1.12)	1.75 (− 0.33; 3.84)	− 2.29* (− 3.95; − 0.63)	0.15 (− 2.13; 2.44)	− 0.17 (− 2.25; 1.89)	0.19 (− 2.35; 2.75)	1.64 (− 0.85; 4.14)	− 0.71 (− 2.57; 1.14)	− 2.79* (− 4.81; − 0.78)
Street connectivity (score 1–4)^2^	− 0.26* (− 0.37; − 0.15)	0.40 (− 1.05; 1.85)	− 0.56* (− 0.68; − 0.44)	1.06 (− 0.43; 2.57)	− 0.86 (− 2.50; 0.77)	− 0.68 (− 2.63; 1.27)	− 0.47 (− 2.45; 1.51)	− 0.24 (− 1.77; 1.29)	− 0.08 (− 1.67; 1.50)
Walking/cycling facilities (score 1–4)^1^	− 0.31 (− 0.81; 0.17)	− 0.71 (− 2.44; 1.01)	0.47 (− 0.68; 1.64)	0.42 (− 1.24; 2.08)	0.55 (− 0.93; 2.03)	0.69 (− 0.79; 2.18)	− 1.14 (− 3.16; 0.88)	0.09 (− 1.03; 1.21)	− 1.94* (− 2.12; − 1.76)
Aesthetics (score 1–4)^1^	0.19 (− 0.24; 0.63)	− 1.66* (− 3.03; − 0.30)	0.64 (− 0.23; 1.52)	− 0.71 (− 2.02; 0.59)	0.74 (− 0.60; 2.09)	− 0.32 (− 1.92; 1.27)	− 0.22 (− 1.79; 1.34)	0.62 (− 0.54; 1.78)	− 0.21 (− 1.43; 0.99)
Safety from traffic (score 1–4)^2^	− 0.79* (− 1.47; − 0.12)	− 2.93* (− 4.73; − 1.13)	− 1.40* (− 1.67; − 1.13)	0.69 (− 1.16; 2.56)	− 0.05 (− 1.82; 1.71)	− 0.88 (− 3.14; 1.38)	− 1.59 (− 3.72; 0.53)	− 1.26 (− 3.14; 0.60)	0.81 (− 1.32; 2.95)
Safety from crime (score 1–4)^1^	− 0.10 (− 0.63; 0.43)	− 0.73 (− 2.39; 0.92)	0.23 (− 0.94; 1.41)	− 1.29 (− 2.96; 0.37)	− 0.48 (− 1.96; 0.99)	0.01 (− 1.87; 1.88)	− 1.66 (− 3.46; 0.14)	− 1.38 (− 3.01; 0.24)	1.29 (− 0.23; 2.81)

Linear regression models adjusted for age, sex, marital status, work status, socioeconomic level, and energy intake; work status, socioeconomic level, and energy intake.

*Indicates statistically significant associations (p < 0.05).

*CI* confidence interval.

^1^Higher scores indicate perception of higher land use mix− diversity, higher land use mix-access, more walking/cycling facilities, better aesthetics, and more safety from crime.

^2^4-point scale: strongly disagree (1), disagree (2), agree (3), strongly agree (4).

Overall, land use mix-diversity (β: − 0.11, 95% CI − 0.20; − 0.03), land use mix-access (β: − 0.23, 95% CI − 0.34; − 0.12), walking/cycling facilities (β: − 0.22, 95% CI − 0.37; − 0.08), and safety from crime (β: − 0.27, 95% CI − 0.42; − 0.12) were negatively associated with neck circumference (cm). When conducting country specific analyses, some distinct associations were identified. For instance, Argentina was the only country where high land use mix-access (β: − 0.25, 95% CI − 0.36; − 0.14), walking/cycling facilities (β: − 0.49, 95% CI − 0.94; − 0.04), and aesthetics (β: − 0.42, 95% CI − 0.78; − 0.07) were associated with less neck circumference (Table 4).

**Table 4:** Linear regression models (unstandardized β, 95% CI) for perceived urban environment attributes and neck circumference (cm).

Independent variables	Overall	Argentina	Brazil	Chile	Colombia	Costa Rica	Ecuador	Peru	Venezuela
	β (95% CI)	β (95% CI)	β (95% CI)	β (95% CI)	β (95% CI)	β (95% CI)	β (95% CI)	β (95% CI)	β (95% CI)
Land use mix-diversity (score 1–5)^1^	− 0.11* (− 0,20; − 0.03)	− 0.05 (− 0,35; 0.23)	− 0.56* (− 0.82; − 0.30)	0.19 (− 0.27; 0.66)	0.27 (− 0.01; 0.55)	0.01 (− 0.35; 0.36)	0.34 (− 0.09; 0.78)	− 0.12* (− 0.20; − 0.04)	0.01 (− 0.29; 0.33)
Land use mix-access (score 1–4)^1^	− 0.23* (− 0.34; − 0.12)	0.25* (− 0.36; − 0.14)	− 0.28 (− 0.81; 0.24)	0.38 (− 0.25; 1.01)	− 0.41 (− 0.98; 0.15)	0.22 (− 0.42; 0.87)	0.59 (− 0.17; 1.36)	0.17 (− 0.38; 0.72)	− 0.23 (− 0.83; 0.35)
Street connectivity (score 1–4)^2^	0.10 (− 0.03; 0.23)	0.30 (− 0.07; 0.68)	− 0.39* (− 0.75; − 0.04)	0.27 (− 0.14; 0.69)	0.04 (− 0.40; 0.49)	0.34 (− 0.15; 0.83)	0.13 (− 0.48; 0.74)	− 0.10 (− 0.35; 0.55)	0.29 (− 0.17; 0.75)
Walking/cycling facilities (score 1–4)^1^	− 0.22* (− 0.37; − 0.08)	− 0.49* (− 0.94; − 0.04)	− 0.30* (− 0.51; − 0.09)	0.01 (− 0.44; 0.47)	− 0.10 (− 0.51; 0.29)	0.02 (− 0.36; 0.39)	− 0.39 (− 1.01; 0.23)	0.04 (− 0.28; 0.38)	0.20 (− 0.19; 0.60)
Aesthetics (score 1–4)^1^	0.09 (− 0.04; 0.21)	− 0.42* (− 0.78; − 0.07)	− 0.10 (− 0.33; 0.17)	− 0.10 (− 0.46; 0.26)	− 0.17 (− 0.28; − 0.06)	0.32 (− 0.08; 0.72)	0.02 (− 0.46; 0.50)	0.25 (− 0.09; 0.59)	0.32 (− 0.02; 0.68)
Safety from traffic (score 1–4)^2^	0.01 (− 0.20; 0.20)	− 0.37 (0.84; 0.10)	− 0.17 (− 0.67; 0.32)	0.43 (− 0.08; 0.95)	0.19 (− 0.28; 0.68)	0.05 (− 0.52; 0.62)	0.08 (− 0.56; 0.74)	0.03 (− 0.52; 0.59)	0.16 (− 0.46; 0.78)
Safety from crime (score 1–4)^1^	− 0.27* (− 0.42; − 0,12)	0.09 (− 0.34; 0.52)	0.30 (− 0.06; 0.68)	− 0.06 (− 0.52; 0.40)	0.18 (− 0.22; 0.59)	0.44 (− 0.33; 0.92)	0.01 (− 0.55; 0.55)	− 0.03 (− 0,51; 0.45)	0.09 (− 0.35; 0.54)

Linear regression models adjusted for age, sex, marital status, work status, socioeconomic level, and energy intake.

*Indicates statistically significant associations (p < 0.05).

*CI* confidence interval.

^1^Higher scores indicate perception of higher land use mix-diversity, higher land use mix-access, more walking/cycling facilities, better aesthetics, and more safety from crime.

^2^4-point scale: strongly disagree (1), disagree (2), agree (3), strongly agree (4).

Overall and by country, no significant associations were observed between the any perceived urban environment attributes and a body shape index (Table 5).

**Table 5:** Linear regression models (unstandardized β, 95% CI) for perceived urban environment attributes and a body shape index (cm).

Independent variables	Overall	Argentina	Brazil	Chile	Colombia	Costa Rica	Ecuador	Peru	Venezuela
	β (95% CI)	β (95% CI)	β (95% CI)	β (95% CI)	β (95% CI)	β (95% CI)	β (95% CI)	β (95% CI)	β (95% CI)
Land use mix-diversity (score 1–5)^1^	− 0.02 (− 0.03; 0.00)	0.00 (− 0.01; 0.01)	0.00 (0.00; 0.01)	− 0.01 (− 0.01; 0.00)	0.00 (− 0.01; 0.00)	0.00 (− 0.01; 0.00)	0.00 (− 0.01; 0.00)	0.00 (− 0.01; 0.00)	0.00 (0.00; 0.00)
Land use mix-access (score 1–4)^1^	− 0.01 (− 0.01; 0.00)	0.00 (− 0.01; 0.01)	0.00 (0.00; 0.01)	0.00 (0.00; 0.00)	0.00 (− 0.01; 0.00)	0.00 (− 0.02; 0.00)	0.00 (− 0.01; 0.00)	0.00 (− 0.01; 0.00)	0.00 (0.00; 0.00)
Street connectivity (score 1–4)^2^	0.01 (− 0.01; 0.02)	0.00 (0.00; 0.00)	0.00 (− 0.01; 0.00)	0.00 (− 0.01; 0.00)	0.00 (− 0.01; 0.00)	0.00 (− 0.01; 0.00)	0.00 (− 0.01; 0.00)	0.00 (− 0.01; 0.00)	0.00 (− 0.01; 0.00)
Walking/cycling facilities (score 1–4)^1^	− 0.01 (− 0.03; 0.01)	− 0.01 (− 0.02; 0.00)	− 0.01 (− 0.02; 0.00)	− 0.01 (− 0.02; 0.00)	0.00 (− 0.01; 0.00)	0.00 (− 0.01; 0.00)	0.00 (− 0.01; 0.00)	0.00 (− 0.01; 0.00)	0.00 (− 0.01; 0.00)
Aesthetics (score 1–4)^1^	− 0.01 (− 0.01; 0.00)	− 0.01 (− 0.01; 0.00)	0.00 (− 0.01; 0.00)	0.00 (− 0.01; 0.00)	0.00 (− 0.01; 0.00)	0.00 (− 0.01; 0.00)	0.00 (− 0.01; 0.00)	0.00 (− 0.01; 0.00)	0.00 (− 0.01; 0.00)
Safety from traffic (score 1–4)^2^	− 0.03 (− 0.04; − 0.02)	0.00 (− 0.01; 0.00)	0.00 (− 0.01; 0.00)	0.00 (− 0.01; 0.00)	0.00 (− 0.01; 0.01)	0.01 (− 0.01; 0.01)	− 0.01 (− 0.02; 0.00)	0.00 (− 0.01; 0.00)	0.00 (− 0.01; 0.00)
Safety from crime (score 1–4)^1^	0.01 (− 0.01; 0.03)	0.00 (− 0.01; 0.00)	0.00 (− 0.01; 0.00)	0.00 (− 0.01; 0.00)	− 0.00 (− 0.01; 0.00)	0.00 (− 0.01; 0.01)	0.01 (− 0.01; 0.00)	0.00 (− 0.01; 0.00)	0.00 (− 0.01; 0.01)

Linear regression models adjusted for age, sex, marital status, work status, socioeconomic level, and energy intake.

*indicates statistically significant associations (p < 0.05).

*CI* confidence interval.

^1^Higher scores indicate perception of higher land use mix-diversity, higher land use mix-access, more walking/cycling facilities, better aesthetics, and more safety from crime.

^2^4-point scale: strongly disagree (1), disagree (2), agree (3), strongly agree (4).

Overall, land use mix-diversity (β: − 0.01, 95% CI − 0.02; − 0.01), aesthetics (β: − 0.02, 95% CI − 0.03; − 0.01), and safety from crime (β: − 0.02, 95% CI − 0.04; − 0.01) were associated with waist-to-height ratio. Distinct associations by country were observed between perceived urban environment attributes and waist-to-height ratio. For instance, aesthetics (β: − 0.03, 95% CI − 0.05; − 0.01), safety from traffic (β: − 0.01, 95% CI − 0.02; − 0.01) and safety from crime (β: − 0.02, 95% CI − 0.03; − 0.01) were associated with waist-to-height ratio in Brazil (Table 6).

**Table 6:** Linear regression models (unstandardized β, 95% CI) for perceived urban environment attributes and waist-to-height ratio.

Independent variables	Overall	Argentina	Brazil	Chile	Colombia	Costa Rica	Ecuador	Peru	Venezuela
	β (95% CI)	β (95% CI)	β (95% CI)	β (95% CI)	β (95% CI)	β (95% CI)	β (95% CI)	β (95% CI)	β (95% CI)
Land use mix-diversity (score 1–5)^1^	− 0.01* (− 0.02; − 0.01)	− 0.01 (− 0.01; 0.00)	− 0.01 (− 0.01; 0.00)	− 0.01 (− 0.01; 0.00)	− 0.01 (− 0.01; 0.00)	− 0.01 (− 0.02; 0.00)	− 0.01 (− 0.02; 0.00)	− 0.01 (− 0.02; 0.00)	− 0.01 (− 0.01; 0.00)
Land use mix-access (score 1–4)^1^	0.01 (− 0.01; 0.02)	0.01 (− 0.02; 0.02)	0.03 (− 0.01; 0.07)	0.03 (− 0.03; 0.06)	− 0.01* (− 0.02; − 0.01)	0.03 (− 0.01; 0.07)	− 0.01 (− 0.01; 0.01)	− 0.01 (− 0.02; 0.01)	− 0.01 (− 0.01; 0.01)
Street connectivity (score 1–4)^2^	0.00 (− 0.01; 0.01)	0.01 (− 0.01; 0.02)	0.01 (− 0.01; 0.01)	0.01 (− 0.01; 0.02)	− 0.01 (− 0.02; 0.01)	0.01 (− 0.01; 0.02)	0.01 (− 0.01; 0.01)	− 0.01 (− 0.02; 0.01)	− 0.01 (− 0.01; 0.01)
Walking/cycling facilities (score 1–4)^1^	0.00 (− 0.01; 0.01)	0.00 (− 0.00; 0.01)	0.00 (− 0.01; 0.01)	0.00 (− 0.00; 0.01)	0.00 (− 0.00; 0.01)	0.00 (− 0.00; 0.01)	0.01 (− 0.00; 0.01)	0.02 (− 0.04; 0.06)	0.01 (− 0.00; 0.01)
Aesthetics (score 1–4)^1^	− 0.02* (− 0.03; − 0.01)	− 0.02* (− 0.03; − 0.01)	− 0.03* (− 0.05; − 0.01)	− 0.04 (− 0.08; 0.00)	− 0.03 (− 0.06; 0.01)	− 0.02 (− 0.04; 0.00)	0.00 (− 0.01; 0.00)	− 0.00 (− 0.01; 0.00)	− 0.00 (− 0.01; 0.00)
Safety from traffic (score 1–4)^2^	− 0.01 (− 0.01; 0.01)	− 0.01* (− 0.02; − 0.01)	− 0.01* (− 0.02; − 0.01)	− 0.00 (− 0.01; 0.00)	− 0.01* (− 0.02; − 0.01)	− 0.00 (− 0.01; 0.00)	− 0.01 (− 0.02; 0.00)	− 0.01 (− 0.02; 0.00)	− 0.01 (− 0.02; 0.00)
Safety from crime (score 1–4)^1^	− 0.02* (− 0.04; − 0.01)	0.00 (− 0.01; 0.00)	− 0.02* (− 0.03; − 0.01)	0.00 (− 0.01; 0.00)	0.01 (− 0.01; 0.00)	− 0.01* (− 0.02; − 0.01)	− 0.01* (− 0.02; − 0.01)	− 0.02* (− 0.03; − 0.01)	− 0.01* (− 0.02; − 0.01)

Linear regression models adjusted for age, sex, marital status, work status, socioeconomic level, and energy intake.

*indicates statistically significant associations (p < 0.05).

*CI* confidence interval.

^1^Higher scores indicate perception of higher land use mix-diversity, higher land use mix-access, more walking/cycling facilities, better aesthetics, and more safety from crime.

^2^4-point scale: strongly disagree (1), disagree (2), agree (3), strongly agree (4).

## Discussion

This study aimed to examine the associations between perceived urban environment attributes and obesity indices in adults from eight Latin America countries. We found that, when participants were pooled together, there was a clear negative relationship between perceived urban environment and obesity indices. For instance, an increased perception of land use mix (β: − 0.44; 95% CI − 0.59; − 0.28), safety from traffic (β: − 0.44; 95% CI − 0.59; − 0.28), and safety from crime (β: − 0.36; 95% CI − 0.57; − 0.15) were associated with a decreased body mass index. The relationship between the built environment and obesity has been vastly explored in the literature^12,15,56^. In this study, we found that an improved perception of the environment in regard to land use mix, traffic safety, and crime safety were associated with a lower body mass index, indicating that a good environment could have the potential to improve health markers and lower obesity indices. Nonetheless, the associations between perceived urban environment attributes and body mass index may be mediated by physical activity. A study of multiple localities found that perceiving diversity of land uses, and safe traffic environments was positively associated with higher levels of moderate to vigorous physical activity^57^.

This study also found that the relationship between perceived environment and lower body mass index was significant only in certain countries. Argentina had the strongest association between the perceived environment and obesity. Brazil and Venezuela showed the same tendency; improved perception of environment associated with lower obesity indices. Countries such as Chile, Colombia, Costa Rica, Ecuador, and Peru did not present the same significance for the variables in this study. Overall, it is known that Latin America countries present high levels of obesity^58^. Latest reports have shown Venezuela (40%), Chile (34%), and Argentina (31%)^59^, to have the highest obesity prevalence, followed by Costa Rica 29%, Colombia 25%, Brazil 22%, Ecuador 22%, and Peru 22%^59^. There is no clear evidence as to why certain countries had an individual association and others did not.

However, some assumptions can be made. Venezuela presents the highest proportion of obese population, which could mean the impact of the perceived neighborhood environment has a wider reach in modulating obesity indices. Argentina has also one of the highest proportions of obesity. Brazil, on the other hand, had the lowest obesity rates and still showed significant results. We hypothesize that this is due to the size of the country, as Brazil is much larger than all the others included in the project and has a much more diverse population and more diverse environments. Though the countries might have distinct attributes in terms of size, population, and characteristics, this study brings value in understanding the overall context of perceived environments and obesity. These perceptions may be of high value when planning interventions that could benefit a wide range of the population. Furthermore, other studies have shown similar results; for example, a study of 12 countries around the world showed associations as the ones reported in the present study, reaffirming that land use mix, traffic safety, and crime safety are three important aspects of the neighborhood environment that should be addressed when using the environment to promote health^17^.

Although the perceived built environment has been associated with obesity, through the anthropometric indices used, with the exception of a body shape index, it is important to highlight the benefits of the social neighborhood environment as well. Once again, physical activity has been identified as a potential lead to the path of association between social environments and obesity^60^.

Historical, political, physical, economic, and social environments not explored by this research might be capable of impacting obesity rates, given that they independently influence environmental relations with obesity indices in the dissimilar countries^61–63^. For instance, Brazil has a dissimilar urban planning and design approach from those of the other countries in the region. Argentina, Colombia, Ecuador, Peru, and Venezuela were mostly colonized by Spain; therefore, their urban morphology is diverse from that of Brazil, which was colonized by Portugal. Although Latin American countries share multiple sociocultural values and characteristics, there are nuances that differentiate them^63,64^. As in most areas of public health, evidence from many countries recommends that policy and environmental strategies will be a vital part of combating obesity rates.

We acknowledge some limitations of this study that included the cross-sectional design which prevents conclusions regarding causality from being made. The use of perceived neighborhood walkability is sometimes considered to be a limitation. On the other hand, previous studies have shown that perceived neighborhood walkability can explain physical activity or body mass index^12,15^. Future manuscripts should examine the mechanisms (perceived neighborhood walkability versus overweight/obesity) by examining how multiple measures of physical activity and sedentary behaviors may mediate the relation between built environments and body mass index.

## Conclusion

Overall, our findings indicate that perceived neighborhood environments were associated with obesity indices such as body mass index, waist circumference, neck circumference and waist-to-height ratio. However, the associations varied across countries and mechanisms that explain these differences have not yet been explored. Though land-use mix, traffic safety, and crime safety are important environmental aspects to be considered in interventions in Latin America countries.

## Data Availability

The datasets generated and/or analyzed during the current study are not publicly available due the terms of consent/assent to which the participants agreed but are available from the corresponding author on reasonable request. Please contact the corresponding author to discuss availability of data and materials.
